# Case Report: The entanglement of infection and autoimmunity: a case of Caplan syndrome

**DOI:** 10.3389/fimmu.2026.1715034

**Published:** 2026-02-25

**Authors:** Zilin Xiong, Yu Wu, Jia Tan, Xinchuan Chen, Jiajia Dong

**Affiliations:** 1 Department of Hematology, West China Hospital, Sichuan University, Chengdu, China; 2 West China Clinical Medical College, Sichuan University, Chengdu, China; 3 West China School of Medicine, West China Hospital, Sichuan University, Chengdu, Sichuan, China; 4 Department of Radiology, Functional and Molecular Imaging Key Laboratory of Sichuan Province, West China Hospital, Sichuan University, Chengdu, China; 5 Department of Pulmonary and Critical Care Medicine, West China Hospital, Sichuan University, Chengdu, China

**Keywords:** autoimmune, Caplan syndrome, infection, pneumoconiosis, rheumatoid arthritis

## Abstract

Caplan syndrome is a rare disorder characterized by the coexistence of pneumoconiosis and rheumatoid arthritis (RA), primarily observed in individuals with silica or coal dust exposure. Data on disease behavior in the context of incomplete or irregular immunosuppressive therapy remain limited. This case report describes a 56-year-old man with a history of mixed dust exposure and smoking who presented with recurrent fever, chest pain, and polyarthralgia. Despite being diagnosed and treated for pulmonary tuberculosis and cryptococcosis, his respiratory and articular symptoms persisted. Serological tests revealed persistently elevated rheumatoid factor (RF) and anticitrullinated peptide antibody (ACPA), while a lung biopsy demonstrated chronic inflammation with carbon deposition, confirming Caplan syndrome. Retrospective analysis indicated that elevations of RF and ACPA preceded observable radiological and pathological changes. Notably, inflammatory markers continued to rise significantly even after infections were controlled and despite only episodic glucocorticoid use. This case highlights the importance of early serological monitoring and comprehensive management in high-risk patients and underscores that, rather than being viewed merely as complications, infections in autoimmune contexts should be recognized as potential triggers and aggravators of immune dysregulation, warranting heightened vigilance in both diagnosis and management.

## 1 Introduction

Caplan syndrome is a rare pulmonary complication of rheumatoid arthritis (RA), defined by the concurrence of multiple necrobiotic nodules in patients with underlying pneumoconiosis, most frequently silicosis (1). Epidemiologic data indicate a prevalence of less than 1% among dust-exposed individuals, underscoring its rarity even within high-risk populations (2). Approximately 40% of affected individuals had no joint symptoms despite seropositivity for rheumatoid factor (RF), and their pulmonary lesions often preceded the onset of arthritis by several years (3). Characteristic findings on chest X-ray include multiple, well-circumscribed nodules measuring 1–2 cm in diameter, typically localized to the peripheral upper lung zones. Confluent nodular growth and cavitation may also occur (3). Histopathology of Caplan nodules is defined by concentrically layered necrosis surrounded by fibrotic tissue containing dust-laden macrophages, granulocytes, and particulate deposits. Detection of inorganic dust particles via polarized microscopy can aid diagnosis, but is not required (1). The pathophysiology of Caplan syndrome remains debated; however, silica exposure and dysregulated immunity pathways are key mediators of its pathogenesis.

This complexity is further compounded by the diagnostic challenge posed by coexisting infections, as illustrated in the present case. A dust-exposed male patient presented with initially masked Caplan syndrome, in whom sequential infections with tuberculosis (TB) and cryptococcosis obscured the underlying pathology. This clinical scenario highlights the diagnostic and pathophysiological complexity arising from the coexistence of environmental particulate exposure, individual susceptibility, and opportunistic infections, underscoring the need to address overlapping etiologies in high-risk populations.

## 2 Case presentation

A 56-year-old man was admitted with a 2-month history of recurrent low-grade fever, chest pain, and generalized arthralgia. These symptoms were transiently relieved by NSAIDs. He had no cough, sputum, hemoptysis, or night sweats.

**Figure 1:**
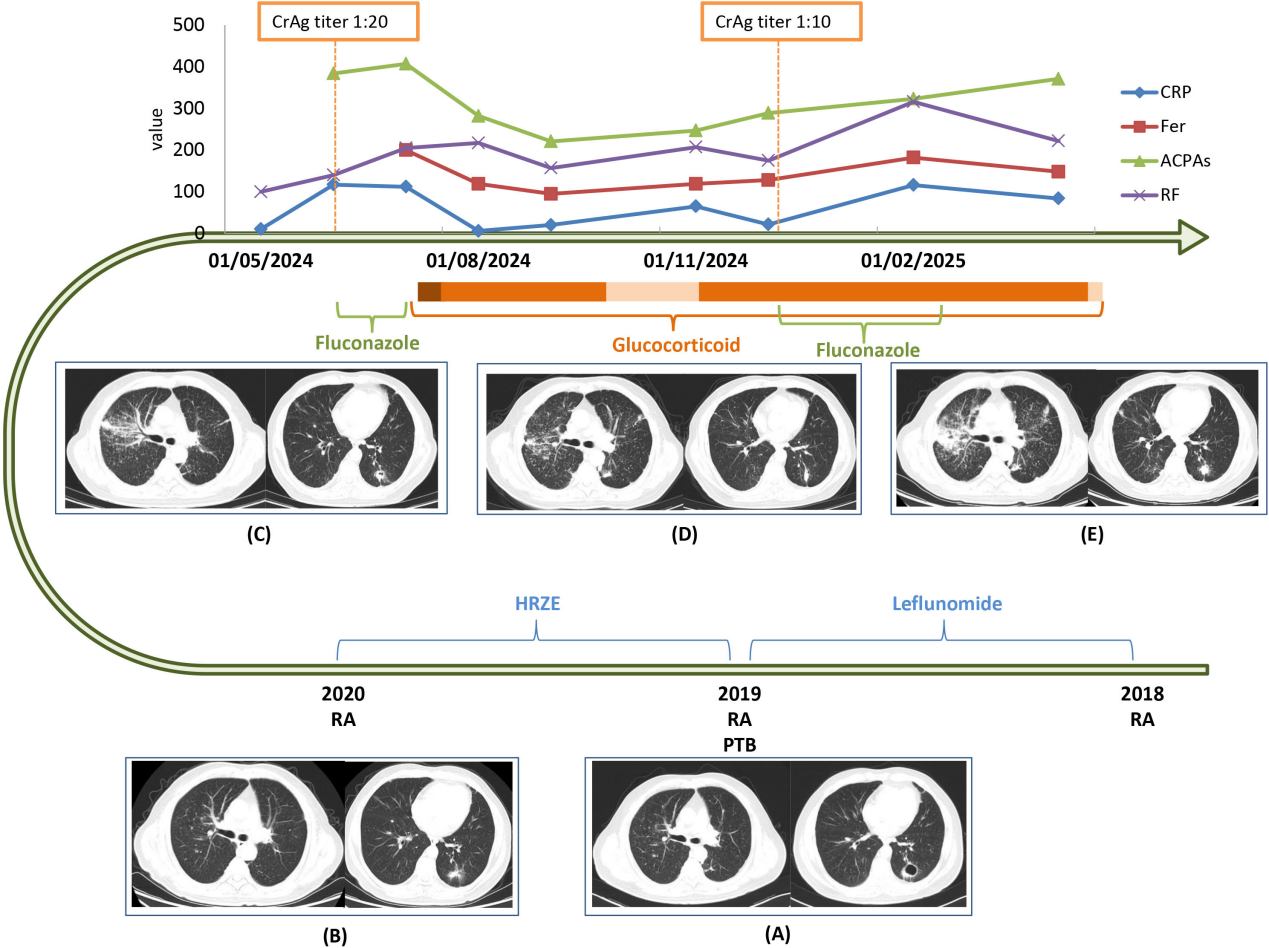
Timeline of major interventions and therapeutic effects, 2018–2025. **(A–E)** Serial chest CT images: **(A)** at onset of anti-TB therapy (HRZE); **(B)** after 12 months of HRZE; **(C)** before antifungal treatment; **(D)** after pulse glucocorticoid treatment; **(E)** at recurrence of the serum CrAg titer. Bottom temporal: evolution of laboratory markers. Blue line, C-reactive protein (CRP; mg/L); red line, ferritin (Fer; × 10 ng/mL); green line, anticitrullinated protein antibodies (ACPA; U/mL); purple line, rheumatoid factor (RF; × 10 IU/mL). Orange bars beneath the *x*-axis indicate glucocorticoid dose: dark orange = 50 mg daily prednisone-equivalent, medium orange = 20 mg/day, light orange = 15 mg/day. Antifungal therapy was initiated when CrAg became positive (June 2024); the antigen was negative after 4 weeks, allowing corticosteroid introduction and tapering. CrAg positivity recurred in December 2024. HRZE, isoniazid, rifampicin, pyrazinamide, ethambutol; PTB, pulmonary tuberculosis; CrAg titer, serum cryptococcal antigen titer.

Six years ago, he was diagnosed with RA. One year later, he was diagnosed with pulmonary TB, located in the dorsal segment of the left upper lobe. He received standard antituberculous therapy (isoniazid, rifampicin, pyrazinamide, ethambutol [HRZE]) for 1 year, after which the pulmonary cavity closed. Leflunomide was commenced at the initial RA diagnosis but discontinued 5 years ago when TB was uncovered; no disease modifying antirheumatic drugs (DMARDs) had been taken since (**Figure 1**).

His occupational history includes 15 years of charcoal grilling activities and various mining activities, including 1 year in coal mining, 1 year in gold mining, 1 year in shale mining, and 6 months in tunneling, all without personal protective equipment. He had a 20-year history of tobacco use and no recent travel history.

**Figure 2:**
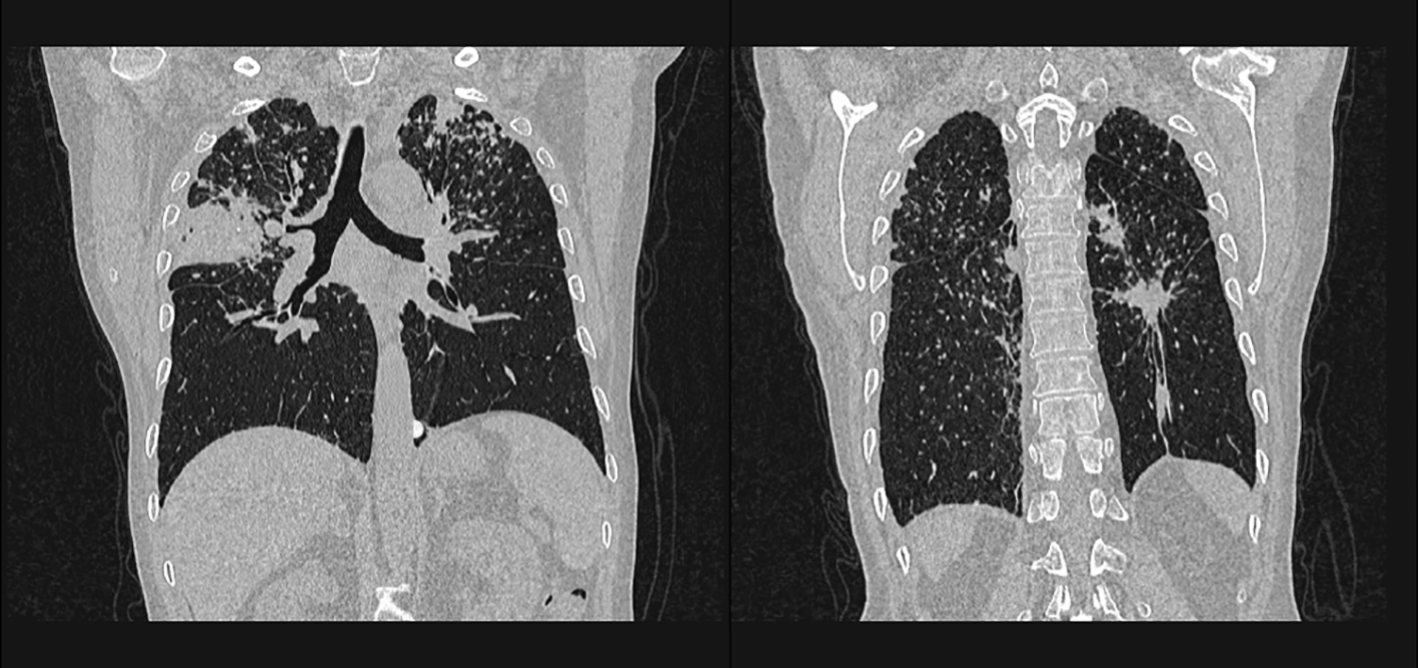
Chest CT on current admission showing the distribution of pulmonary lesions.

On initial physical examination, he was febrile (37.9°C). Fine crackles were noted on auscultation over the right chest. The upper limb joints showed a restricted range of motion due to pain. Chest CT revealed bilateral, multiple subpleural nodules with air bronchograms, predominantly in the upper lobes, along with segmental bronchial wall thickening. The previous TB cavitary lesion located in the left upper lobe had recurred (**Figure 2**).

**Figure 3:**
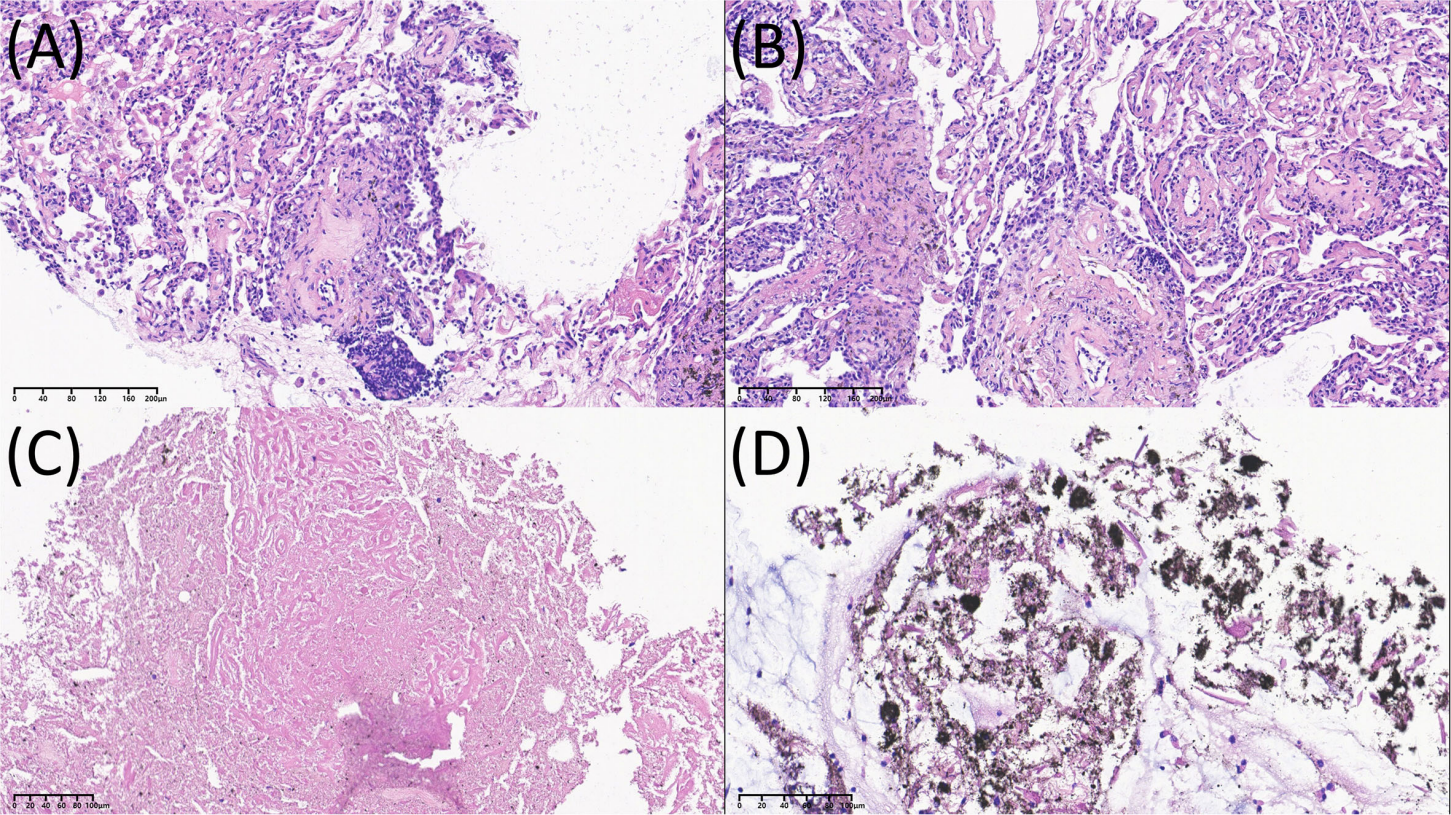
Histopathology of CT-guided lung biopsy and hilar lymph node tissue. **(A, B)** Lung parenchyma; **(C, D)** peribronchial lymph nodes.

Initial laboratory tests showed elevated inflammatory markers (C-reactive protein [CRP], ESR, ferritin), positive RF (2,050 IU/mL) and anti-CCP antibodies (ACPAs; 407 U/mL), but negative ANA/ENA/anti-SSA/anti-SSB/IgG4. The serum cryptococcal antigen (CrAg) titer was positive at 1:20. Tumor markers (CEA, CYFRA21-1, NSE) were within normal limits. Bronchoalveolar lavage (BAL) and central nervous fluid (CSF) showed no pathogens, including acid-fast bacilli, via smear, culture, metagenomic next-generation sequencing (mNGS), and nucleic acid amplification testing (NAAT). Cytology revealed no malignant cells but numerous macrophages laden with black carbonaceous pigment. Furthermore, a CT-guided lung biopsy of the right lobe was performed to obtain histopathological confirmation. It revealed alveolar wall fibroplasia and chronic inflammation with infiltration of numerous lymphocytes and plasmocytes, as well as abundant carbon dust deposition (**Figure 3**). Polarized microscopy and special stains (hexamine silver stain, acid-fast stain, and periodic acid–Schiff stain) were negative. No evidence of tumor or microorganism was found. Notably, the full zonal architecture of a classic Caplan nodule was not captured due to the limited biopsy size, and the central zone could not be fully evaluated microscopically. However, the findings were consistent with a dust-associated inflammatory granulomatous process.

Although 2 weeks of fluconazole rendered the serum CrAg titer negative, systemic inflammation persisted, and intravenous methylprednisolone 40 mg daily (50 mg prednisone-equivalent) was started, leading to rapid improvement of arthralgia, pulmonary lesions, and inflammatory markers (**Figure 1D**). Upon tapering to 15 mg prednisone-equivalent, arthralgia and CRP rebounded; the dose was therefore increased to 20 mg, resulting in symptom restabilization. Subsequently, CrAg rose to 1:10, accompanied by fever and imaging progression (**Figure 1E**). In view of ongoing uncertainty regarding active infection, the introduction of conventional synthetic DMARDs was considered unsafe. Consequently, the patient remains on a maintenance dose of 20 mg/day of prednisone-equivalent, as glucocorticoid withdrawal has proven unachievable.

## 3 Discussion

In the present case, a man with RA and treated pulmonary tuberculosis presented with fever, polyarthralgia, and multiple nodules on chest CT. Although serum CrAg testing was negative, persistent inflammation following an antifungal course argued against infection as the sole driver of disease progression. Histopathology showed nonspecific chronic inflammation. The presence of carbon-laden macrophages, together with a history of dust exposure over 18 years, supported a diagnosis of Caplan syndrome—a rare pneumoconiotic complication of RA.

The diagnostic challenge in this case stemmed from the overlapping features of infection, autoimmunity, and environmental lung disease. The recurrent cavitary lesion and positive CrAg titer initially directed suspicion toward infectious etiologies; however, microorganism tests (culture and smear) and molecular assays (including NAAT and mNGS) showed no evidence of *Mycobacterium tuberculosis complex*, *non-Mtb tuberculosis*, or *invasive fungi*. Indeed, both *Mycobacterium tuberculosis* and *Cryptococcus* can disrupt immune tolerance via molecular mimicry and innate immune activation (4, 5). In this context, we hypothesize that recurrent or persistent pulmonary infections may facilitate the development of Caplan nodule formation in a susceptible host.

RA-related lung disease includes interstitial disease (RA-ILD), airway obstruction, and rheumatoid nodules. In our patient, the absence of ground-glass opacities, reticulation, or honeycombing excluded RA-ILD. Similar to RA-ILD, Caplan nodules are preceded by airway and alveoli damage, which leads to the development of lymphoid follicles and local production of inflammatory cytokines and ACPAs (6). The critical difference lies in distribution and morphology: silica’s aerodynamic preference for the upper and middle lobes directs the ensuing immune response toward nodular fibrosis, whereas the basal-predominant interstitial pattern of RA-ILD reflects a more diffuse, subpleural fibroblast activation. This distinction may be attributed to the fact that Caplan nodules are driven by inhaled silica, which activates local fibroblasts and the NLRP3 inflammasome to create a proinflammatory milieu (7). The key discriminator between simple pulmonary rheumatoid nodules and Caplan nodules is the pattern of dust deposition, best inferred from a detailed occupational history.

Several limitations warrant acknowledgment. First, lung function testing was not performed because the patient was clinically unstable, making the assessment unreliable. Second, the biopsy sample was limited, preventing more detailed immunohistochemical or molecular analyses. Third, the response to glucocorticoid therapy was suboptimal, which may reflect advanced fibrotic change, ongoing microbial stimulation, or incomplete immunosuppression. These factors underscore the challenges in managing such complex, multifactorial lung disease.

In conclusion, this case illustrates how occupational dust exposure, autoimmunity, and recurrent infection may converge to drive pulmonary pathology in RA patients. Positive serological or molecular findings should be interpreted cautiously and in context, as they do not necessarily indicate active infection. Histopathology remains essential for diagnosis when possible. Caplan syndrome should be considered in the differential diagnosis, even when some infectious workup results are positive. The case also emphasizes the importance of obtaining a detailed occupational history in patients with RA and pulmonary nodules. Environmental exposures warrant greater attention in future studies of RA-related lung disease.

## Data Availability

The raw data supporting the conclusions of this article will be made available by the authors, without undue reservation.
